# The Utility of Macrolide Therapy for Neutrophilic CRSsNP Based on Structured Histopathology

**DOI:** 10.1002/lary.70606

**Published:** 2026-05-06

**Authors:** Alison J. Yu, Sanjena Venkatesh, Jadyn Wilensky, Alan D. Workman, Jeremy Chang, Maria Espinosa, Jennifer E. Douglas, James N. Palmer, Nithin D. Adappa, Michael A. Kohanski

**Affiliations:** ^1^ Division of Rhinology, Department of Otorhinolaryngology University of Pennsylvania Philadelphia Pennsylvania USA; ^2^ Division of Rhinology and Sinus Surgery, Department of Otolaryngology ‐ Head and Neck Surgery Medical University of South Carolina Charleston South Carolina USA; ^3^ Perelman School of Medicine, University of Pennsylvania Philadelphia Pennsylvania USA; ^4^ Department of Otolaryngology Massachusetts Eye and Ear/Harvard Medical School Boston Massachusetts USA; ^5^ Ohio State University, College of Medicine Columbus Ohio USA

**Keywords:** azithromycin, chronic rhinosinusitis, CRS endotypes

## 1 Introduction

Macrolide therapy is of interest in chronic rhinosinusitis without nasal polyp (CRSsNP) based on their anti‐inflammatory effects treating neutrophilic lower airway conditions [1, 2, 3]. However, the specific indications for macrolide therapy as an adjunctive treatment for refractory CRSsNP are not well‐defined [4, 5]. Structured histopathology at the time of surgery may help guide postoperative therapy [6]. A recent study showed that neutrophilic infiltrates on structured histopathology were associated with a favorable response to macrolides, but the outcomes of long‐term low dose macrolides in neutrophilic CRSsNP have not been systematically investigated [7].

This was a retrospective cohort study of adult postsurgical CRSsNP patients with neutrophilic infiltrate, defined as either focal or diffuse based on established structured histopathology criteria, between 2019 and 2024 [1, 6]. Subjects were grouped by whether they received at least a 3‐month course of azithromycin therapy (250 mg Monday, Wednesday, and Friday). Treatment decision was made independent of the structured histopathology and was directed when there was a persistent infectious or inflammatory state on endoscopy that was refractory to topical and oral antibiotics and steroids. Macrolide therapy was further continued if there was symptomatic or endoscopic improvement in edema, purulence, or crusting. CRSsNP was diagnosed per the 2021 International Consensus Statement on Rhinosinusitis criteria [4]. Patients with diagnosed immunodeficiency or cystic fibrosis were excluded. All surgeries involved complete bilateral sinus surgery with at least Draf IIa frontal sinusotomy. Postoperative steroids were per practice protocol, with an oral prednisone regimen of 20 mg daily for 2 weeks followed by a taper over approximately 2–3 weeks. All patients were transitioned to topical steroid irrigations within 1–2 weeks of surgery. All macrolide‐treated patients had pre‐treatment EKG to rule out prolonged QT.

For the macrolide group, SNOT‐22 scores were collected pre‐macrolide and during the first 9 months of continuous therapy. Additional oral antibiotic and steroid uses of the macrolide group were compared with those of the postsurgical neutrophilic CRSsNP patients who never received macrolides. To determine the utility of early postoperative macrolide therapy, outcomes of the macrolide group were compared against those measured in the first 9 months postoperatively of the never macrolide group. Multiple logistic regression models were adjusted for potential confounders. Results with *p* < 0.05 were statistically significant. All reported *p*‐values were two‐tailed. Statistical analyses were performed using SPSS version 29 (IBM, New York).

77 postsurgical neutrophilic CRSsNP patients were identified, with 34 (44.2%) in the macrolide group (Table 1). Majority (73.0%) of the macrolide therapy were started within 9 months postoperatively. Mean duration of macrolide therapy was 9.0 ± 6.0 months. The macrolide group had higher Lund‐Mackay scores (13.6 ± 5.8 vs. 8.2 ± 5.6, *p* < 0.001) and revision surgery rates (59.5% vs. 30.0%, *p* = 0.009) than the never macrolide group. Both groups had similar proportions of subjects with ≥ 10 eosinophils per high‐power field (27.0% vs. 25.0%, *p* = 0.839).

**TABLE 1:** Comparisons of baseline characteristics and the need for additional antibiotic and steroid treatments between the macrolide and never macrolide groups.

	Total *n* = 77	Macrolide *n* = 37 (48.1%)	Never macrolide *n* = 40 (51.9%)	*p*
Age (years), mean (SD)	57.7 (14.9)	60.9 (13.3)	54.8 (15.8)	0.070
Female, *n* (%)	42 (54.5%)	18 (48.6%)	24 (60%)	0.320
Race, *n* (%)				0.330
White	67 (87.0%)	34 (91.9%)	33 (82.5%)	
Black	3 (3.9%)	0 (0%)	3 (7.5%)	
Other/Unknown	7 (9.1%)	3 (8.1%)	4 (10.0%)	
Revision surgery, *n* (%)	34 (44.2%)	22 (59.5%)	12 (30.0%)	0.009
Allergy, *n* (%)	56 (72.7%)	25 (67.6%)	31 (77.5%)	0.330
Asthma, *n* (%)	34 (44.2%)	18 (48.6%)	16 (40%)	0.450
Lund‐Mackay Score, mean (SD)^a^	10.7 (6.3)	13.6 (5.8)	8.2 (5.6)	< 0.001
Degree of neutrophilic infiltrate, *n* (%)				0.907
Focal	69 (89.6%)	33 (89.2%)	36 (90.0%)	
Diffuse	8 (10.4%)	4 (10.8%)	4 (10.0%)	
Eosinophils, *n* (%)				0.839
< 10	57 (74.0%)	27 (73.0%)	30 (75.0%)	
≥ 10 per HPF	20 (26.0%)	10 (27.0%)	10 (25.0%)	
Additional oral antibiotics, *n* (%)				
0–3 months	15 (19.5%)	3 (8.1%)	12 (30.0%)	0.015
3–6 months^b^	14 (21.5%)	3 (9.7%)	11 (32.4%)	0.026
6–9 months^c^	10 (20.8%)	1 (5.9%)	9 (29.0%)	0.059
Oral steroids, *n* (%)				
0–3 months	11 (14.3%)	3 (8.1%)	8 (20.0%)	0.136
3–6 months^b^	5 (7.7%)	1 (3.2%)	4 (11.8%)	0.197
6–9 months^c^	7 (15.6%)	0 (0%)	7 (22.6%)	0.034

Abbreviations: HPF, high power field; SD, standard deviation.

^a^ Data available for 34 patients in the macrolide group and 40 patients in the never macrolide group.

^b^ Data available for 65 patients.

^c^ Data available for 48 patients.

Additional oral antibiotic use was significantly decreased in the macrolide group up to 6 months (< 3 months: 8.1% vs. 30.0%, *p* = 0.015; 3–6 months: 9.7% vs. 32.4%, *p* = 0.026). Oral corticosteroid use was significantly lower in the macrolide group at 6–9 months (0% vs. 22.6%, *p* = 0.034). After adjusting for preoperative Lund‐Mackay score, revision surgery, and eosinophilic count, macrolide use remained significantly associated with lower antibiotic use < 3 months (adjusted OR: 0.20 [0.04–0.97], *p* = 0.046) and trended toward significance for lower antibiotics use at 3–6 months (adjusted OR: 0.21 [0.04–1.01], *p* = 0.051). Baseline pre‐macrolide SNOT‐22 for the macrolide group was not significantly different than the presurgery SNOT‐22 of the never macrolide group (29.3 ± 17.8 vs. 37.6 ± 21.0, *p* = 0.072). Following the initiation of macrolides, subjects had a mean SNOT‐22 of 12.5 ± 12.1 at 9 months, whereas those in the never macrolide group had a mean SNOT‐22 of 20.3 ± 8.1 at 9 months following surgery (*p* = 0.099).

## 2 Discussion

In CRSsNP patients with neutrophils on structured histopathology, long‐term low dose macrolide therapy after surgery was associated with decreased additional oral antibiotic and steroid uses compared to those never on macrolides, suggesting a possible role for macrolides in the early postoperative period. Previous studies on the utility of macrolides to treat refractory CRS showed mixed results, which could be attributed to the heterogenous patient populations including various CRS endotypes [5, 8, 9]. The observed benefit of macrolides in CRSsNP subjects with neutrophilic inflammation may be due to the inhibition of IL‐8‐mediated neutrophil migration, suggesting a possible role in targeting those with a type‐1 or type‐3 endotype [10, 11].

No subject in this study experienced major gastrointestinal or cardiac adverse effects, suggesting the safety and tolerability of long‐term macrolide treatment. This study has limitations inherent to its retrospective and preliminary nature, including the variable timing and initiation of macrolide therapy that was determined at the discretion of the treating physicians. Additional controlled trials are warranted to further clarify the role of macrolides in the postoperative period for this group of refractory CRSsNP.

## 3 Conclusion

Structured histopathology may be used to help guide early macrolide therapy after surgery for CRSsNP driven by neutrophilic inflammation.

## Funding

The authors have nothing to report.

## Disclosure

The authors have nothing to report.

## Conflicts of Interest

The authors declare no conflicts of interest.

## Data Availability

The data that support the findings of this study are available on request from the corresponding author. The data are not publicly available due to privacy or ethical restrictions.
